# Supplementary material for: Multi-year presence of humpback whales in the Atlantic sector of the Southern Ocean but not during El Niño
Source: Commun Biol. 2021 Jun 25;4:790. doi: 10.1038/s42003-021-02332-6 (PMC8233365; doi:10.1038/s42003-021-02332-6)
Supplement: Supplementary file 2 — Supplementary material [file 42003_2021_2332_MOESM2_ESM.pdf]

Multi-year presence of humpback whales in the Atlantic sector of the Southern Ocean but not during El Niño

- Supplementary Information -

Authors

Elena Schall, Karolin Thomisch, Olaf Boebel, Gabriele Gerlach, Sari Mangia Woods, Ahmed El-Gabbas, Ilse Van Opzeeland

Supplementary Note 1

Material and Methods

Automatic detection and classification of humpback whale vocalizations

All available passive acoustic data were processed by the 'Low frequency detection and classification system' (LFDCS) developed by Baumgartner and Mussoline [1] in order to automatically detect and classify humpback whale vocalizations. A call library for humpback whale call types from the Atlantic sector of the Southern Ocean was constructed using data from two recording periods with confirmed humpback whale acoustic presence: May 2011 and June 2013 from recorders deployed at 59°S 0°E, 64°S 0°E, and 61°S 55°W. In total seven common humpback whale call types were included in the call library, comprising between 153 and 332 selected exemplars (Table *1*). To avoid the miss-classification of vocalizations from other marine mammal species (inhabiting the wider Weddell Sea area and occupying a similar frequency range as humpback whales) as a humpback whale vocalization, at least one common call type per species was determined to be included in the call library. In total seven additional call types from other vocal marine mammal species were included in the call library with between 160 and 321 selected exemplars per call type (Table *1*; [2-5]). The humpback whale call type 18, a low frequency downsweep (*LF DS*), has acoustic characteristics that were very similar to those of the common low frequency downsweeps of other baleen whale species [6-8]. For this reason, the automatic detections of this particular call type cannot be considered as a reliable sign for humpback whale acoustic presence and corresponding detections were therefore only considered in combination with other humpback whale call type detections.

Table 1. LFDCS call library of all tonal sounds which serve as choices for the classification algorithm. Call type numbers were assigned arbitrarily (Humpback whale call type numbers were chosen to match the call type number given in catalogue for manual analysis). Call type names were assigned, based on the visual and aural appearance of the call types during analysis (e.g., 'LF' = low frequency; 'DS' = downsweep).

Species	Call Type	Name	N exemplars
**Humpback whale**	1	*Moan*	200
	3	*Roof*	192
	4	*J*	270
	5	*L*	191
	6	*MoanUp*	166
	18	*LF DS*	153
	19	*LF Moan*	332
**Minke whale**	30	*Bioduck call*	213
**Killer whale**	31	*Excited DS*	268
**Weddell seal**	32	*Long DS*	173
**Crabeater seal**	33	*Low Moan*	160
**Leopard Seal**	34	*Low trill*	275
**Leopard Seal**	35	*High trill*	139
**Ross Seal**	36	*Sirene call*	321

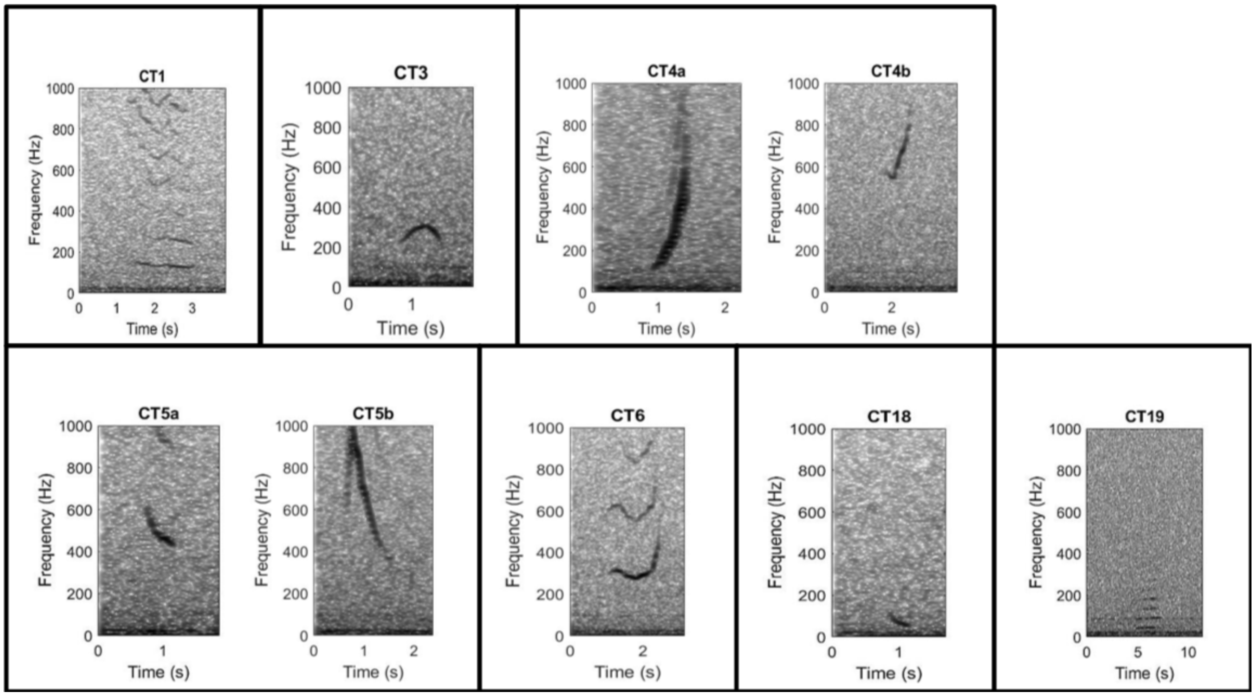

Figure 1. Exemplary humpback whale vocalizations of the call library.

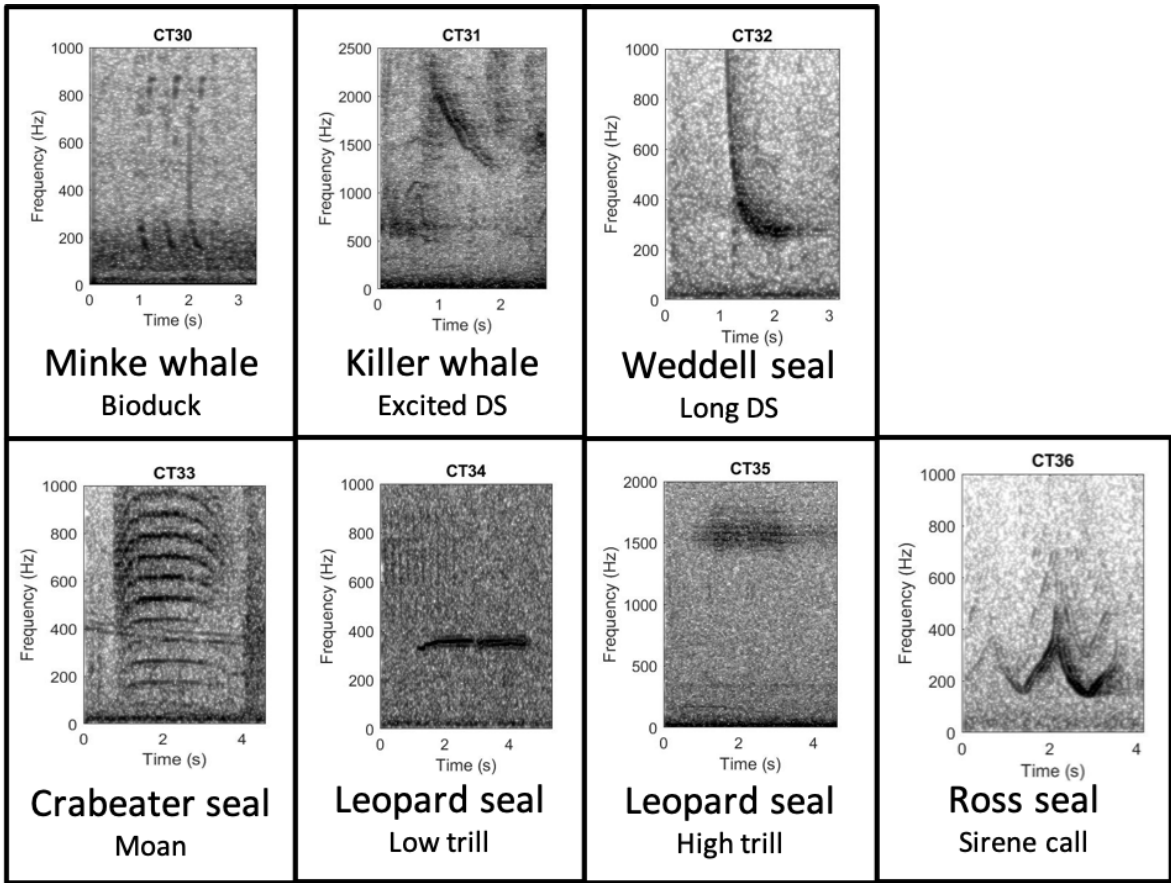

Figure 2. Exemplary marine mammal vocalizations of the call library. Call type names were chosen, based on the visual and aural appearance of the call types during analysis ('DS' = downsweep).

In order to tune the LFDCS detection and classification parameters to yield the best possible detector/classifier performance, a two-step evaluation analysis was applied to selected subsets of acoustic recordings. The first evaluation step was performed on a subset of the passive acoustic data summing up to 30 recording hours (30h-dataset). The 30h-dataset was selected in order to contain different quality humpback whale social calls and songs in different noise conditions (i.e., environmental, anthropogenic and electronic), silent periods, periods with only noise and periods with vocalizations from other marine mammals (i.e., Antarctic blue whale, fin whale, Antarctic minke whale, sperm whale, killer whale, leopard seal, Ross seal, crabeater seal and Weddell seal). This 30h-dataset was compiled from six different recording locations, three different years and all four different seasons. The 30h-dataset was manually screened in Raven Pro 1.5 (Hann Window, 1025 window size, 80% overlap, 2048 DFT size; Bioacoustics Research Program 2014) by marking the start-time of each clearly assignable humpback whale vocalization. Manually detected vocalizations were manually classified into the seven tonal humpback whale call types included in the LFDCS call library. Further, the 30h-dataset was repeatedly

automatically processed in LFDCS using the above mentioned customized call library with detection and classification parameters changing between single LFDCS runs in order to determine the optimal parameter settings (23 adjustable parameters; see Baumgartner and Mussoline [1] for parameter descriptions). Parameter settings were optimized in a parameter optimization cycle, which cycled through 2400 LFDCS runs with randomly chosen parameter combinations. For each run, automatic humpback whale call detections were compared against manual humpback whale call detections with a start-time buffer of 1.8 s (validated via manual comparison of detection start-times in the LFDCS browse mode). The number of true positive, false positive and false negative detections was determined and used to calculate recall, precision and ultimately F1 score [9].

The second evaluation step was designed to evaluate detection efficiency on an hourly basis. For this purpose, ten different parameter settings were chosen from the optimization cycle runs based on their step one performance results, i.e. the balance between recall, precision and F1 score (Figure *3*). Step two of the evaluation procedure was performed on a subset of the passive acoustic data summing up to 150 recording hours (150h-dataset) compiled from the same six locations, three years and four seasons as the 30h-dataset. Likewise, the 150h-dataset was composed of recordings with similar variable acoustic conditions as the 30h-dataset (i.e., including different noise conditions and vocalizations of the different marine mammal species). The 150h-dataset was manually screened in Raven Pro 1.5 (Hann Window, 1025 window size, 80% overlap, 2048 DFT size; Bioacoustics Research Program 2014) by detecting humpback whale acoustic presence on an hourly basis. In LFDCS, the 150h-dataset was processed in ten runs, each with one of the parameter settings chosen from the first evaluation step. For each run, automatic humpback whale detections were compared against manual detections of humpback whale acoustic presence per hour. To minimize false positive hours due to confusion with other species' vocalizations an additional acoustic-context filter was applied before evaluating the results. This acoustic-context filter was based on two conditions: (1) When the number of good quality detections (i.e., Mahalanobis distance (MD) ≤ 2 and signal-to-noise ratio (SNR) ≥ 14) of another species' call type similar to a humpback whale call type (Table *2*) within a respective hour exceeds an hourly call rate (CR) threshold (i.e., 4 calls per hour), and (2) when the number of humpback whale good quality detections (i.e., MD ≤ 2 and SNR ≥ 14; summing over all call types), within a respective hour is lower than an hourly CR threshold (i.e., 6 calls per hour). In case these two conditions were met, all detections of the humpback whale call type similar to the respective other species' call type were deleted from the respective hour.

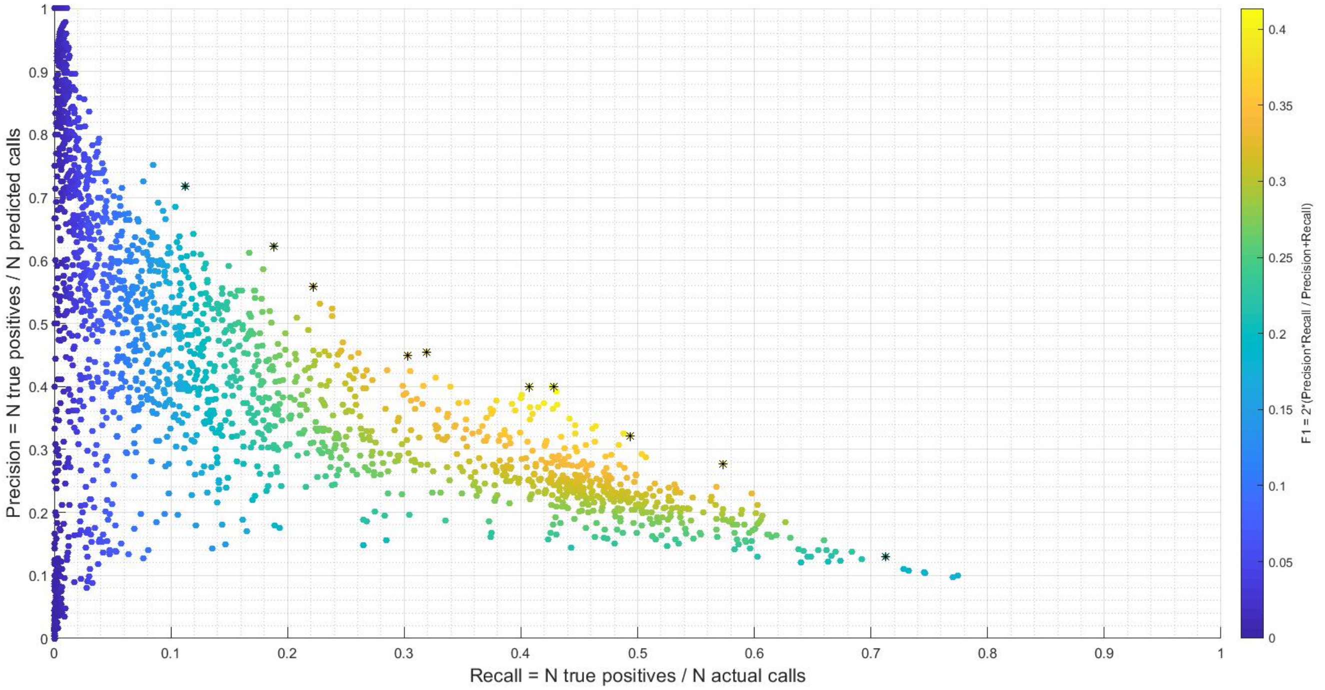

Figure 3. Performance evaluation of 2400 LFDCS runs on the 30h-dataset during step one of the performance evaluation. Ten distinct parameter settings with differently balanced performances (marked with black stars) were chosen for the second evaluation step.

Table 2. Potential sources of miss-identification of humpback whale calls with other species' call types. Humpback whale call types in the first column were frequently mistaken (by LFDCS) for call types of other species as listed in the second column ('CT' = call type).

**Humpback whale call type**	**Similar call type from other species**
**CT1**	leopard seal *Low trill* (CT34), Crabeater seal *Low Moan* (CT33)
**CT3**	Ross seal *Sirene call* (CT36)
**CT5**	killer whale *Excited DS* (CT31), Weddell seal *Long DS* (CT32)
**CT6**	Leopard seal *Low trill* (CT34)
**CT18**	Antarctic minke whale *Bioduck* call (CT30)

Remaining humpback whale call detections were the basis for step two of the evaluation procedure, where hourly detection efficiency of LFDCS and the acoustic context filter was estimated applying different detection quality and hourly CR thresholds. In total seven MD (i.e., 1.5-4.5), seven SNR (i.e., 8-14dB) and 30 CR thresholds (i.e., 1-30) were tested, summing up to 1470 threshold combinations. For each

threshold combination the probability of HW hourly presence ($Prob_{Pres}$) and the probability of false negative hours ($Prob_{FN}$) were calculated:

$$\mathrm{Prob_{Pres}} = \frac{n_{HW\,TP\,hours}}{n_{HW\,predicted\,hours}},$$

$$\mathrm{Prob_{FN}} = \frac{n_{HWFN\,hours}}{n_{HW\,negative\,predicted\,hours}},$$

with the number of humpback whale true positive hours ($n_{HW\,TP\,hours}$), the number of humpback whale false negative hours ($n_{HWFN\,hours}$), the number of humpback whale positive predicted hours ($n_{HW\,predicted\,hours}$), and the number of humpback whale negative predicted hours ($n_{HW\,negative\,predicted\,hours}$). The parameter setting and MD/SNR/CR threshold combination of the run with the highest respective $Prob_{Pres}$ at a $Prob_{FN}$ lower than 20% was finally selected to process the full dataset (Table 3; see Baumgartner and Mussoline [1] for parameter descriptions). Resulting automatically detected hours with presumed humpback whale acoustic presence will be termed presumed *humpback whale presence* (pHWP) hours in the following.

Table 3. Final LFDCS parameter settings and Mahalanobis Distance (MD)/Signal-to-Noise Ratio (SNR)/Call Rate (CR) threshold combination. For parameter descriptions see Baumgartner and Mussoline [1].

**Parameter**	**Value**	**Parameter**	**Value**
Frame	1700 samples	BB_DetectionThreshold	75dB
Overlap	95%	BB_MinSegmentSpan	10Hz
SpectrogramDuration	20s	BB_MinTotalSpan	400Hz
PitchTrackingWindow	15s	BB_MinBroadbandDuration	0.2s
NoiseReductionsWindow	45s	DetectionThreshold	8dB
AvgFFTLowThreshold	-999dB	CostGradientThreshold	15dB
AvgFFTHighThreshold	79dB	DistanceWeighting	30dB
AvgFFTDurationLimit	110s	MinCallDuration	0.3s
BBP_InThreshold	13.5dB	MinAvgAmplitude	8dB
BBP_InDuration	5s	BlankingTime	0.2s
BBP_OutThreshold	5dB	BlankingFreq	2Hz
BBP_OutDuration	0.6s	MD	≤ 2.5
BBP_MaxDuration	25s	SNR	≥ 13dB
		CR	≥ 10

Comparative SNR measurements

In order to evaluate the influence of the missed humpback whale detections for the resulting acoustic presence data of humpback whales in the wider Weddell Sea area, comparative SNR measurements were conducted. Both detected and missed humpback whale calls of the 150h-dataset from the LFDCS run using the final chosen parameter settings (chosen by the highest $Prob_{Pres}$ and a $Prob_{FN}$ lower than 20%) were identified to measure their SNR. In all false negative hours, humpback whale calls were manually identified in Raven Pro 1.5 and their SNRs were measured by comparing the average power (dB re 1µPa) of the spectrum with the call's duration and bandwidth with the average power of two spectra with the same dimensions, one before and one after the call, respectively. From all true positive hours, a number of hours was randomly chosen in order to match the number of false negative hours. Three detected calls were randomly chosen from each of these selected true positive hours and SNRs of these calls were measured applying the same method as described above.

Results

Detector/classifier performance evaluation

In the 30h-dataset, 5274 humpback whale vocalizations were manually detected and classified into the seven humpback whale call types included in the LFDCS call library, with 357 manual detections of call type 1, 439 of call type 3, 2471 of call type 4, 1056 of call type 5, 508 of call type 6, 338 of call type 18, and 100 of call type 19. Different parameter settings in LFDCS yielded different performance results in terms of recall, precision and F1-score (*Figure 3*). The selected parameter setting resulted in a recall of 22%, a precision of 56%, and a F1-score of 0.32. In the present study, for the detection of humpback whale presence on an hourly basis, it was considered more important to aim for a higher precision of the automated detector at the cost of a lower recall (because it was not necessary to detect all vocalizations in order to capture hourly acoustic presence). The final parameter setting in combination with the acoustic-context filter and specific MD, SNR and CR thresholds resulted in a good detection performance in terms of hourly humpback whale presence (Figure *4*). At an hourly CR threshold of at least 10 calls/hour the automatic detection process yielded a $Prob_{Pres}$ of 75% and a $Prob_{FN}$ of only 18%. Because it is common practice to exclude vocalizations with a SNR below 10 dB from the analysis [10,11], the quality of missed vocalizations in these 18% of false negative hours was checked. In comparison to the sampled humpback whale vocalizations in the detected hours, the vocalizations in the false negative hours had SNRs which were mainly below 10 dB (Figure *5*).

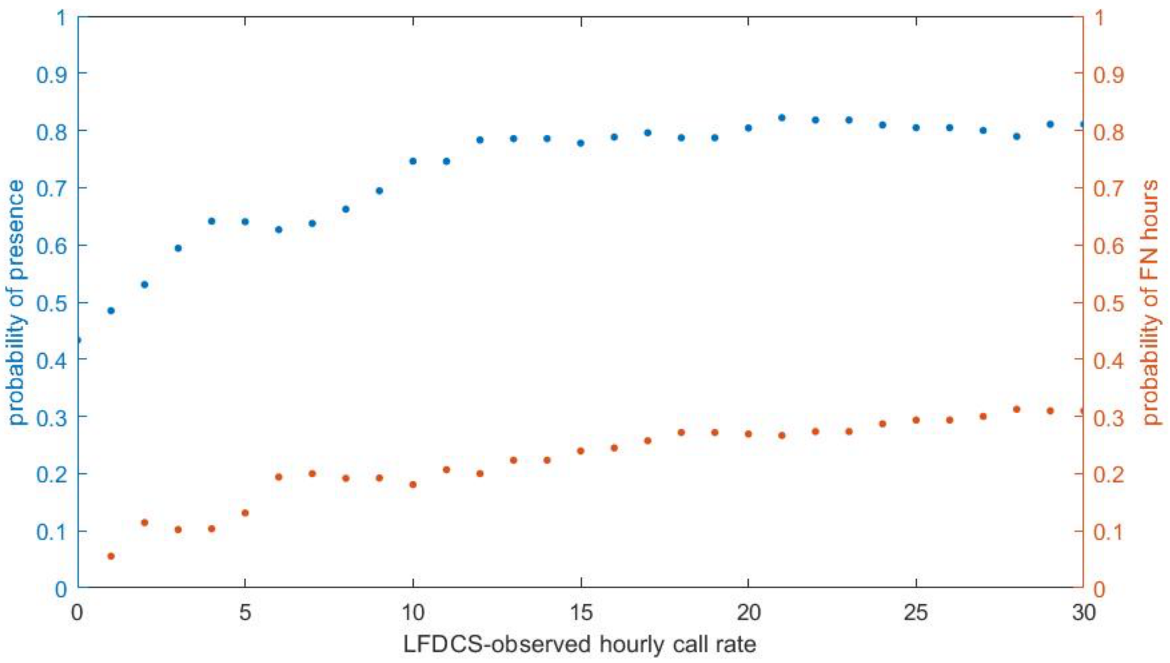

Figure 4. Final detection performance of hourly humpback whale acoustic presence in the 150h-dataset. The probability of humpback whale acoustic presence in the recording hours is depicted in blue on the left y-axis and the probability of false negative hours is depicted in orange on the right y-axis. The x-axis represents the hourly call rate observed by the detector after applying the acoustic-context filter.

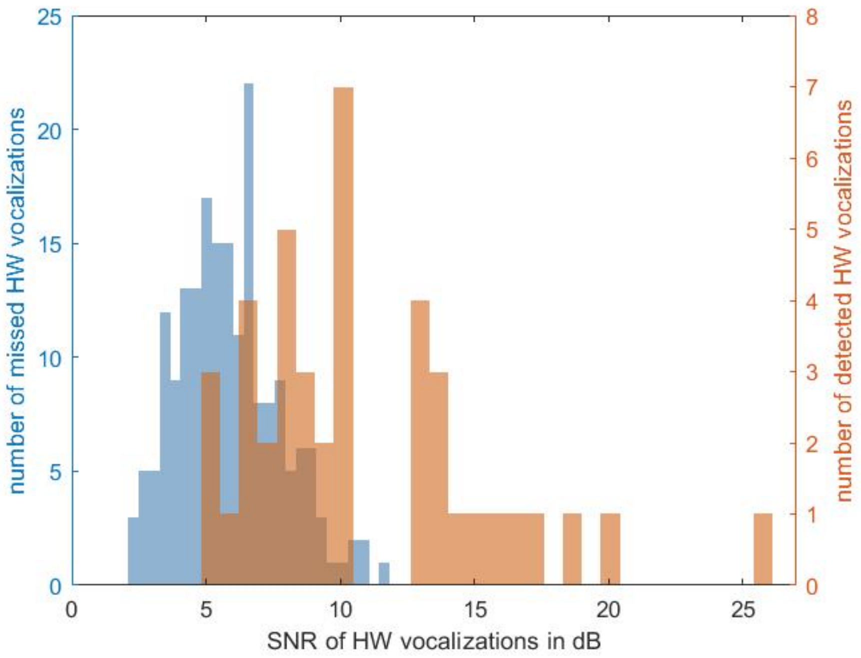

Figure 5. Measured signal-to-noise ratios (SNR) of humpback whale (HW) vocalizations. Blue bars (left y-axis) represent SNRs of vocalizations in false negative (missed) hours and orange bars (right y-axis) represent SNRs of vocalizations in detected hours.

Supplementary Note 2

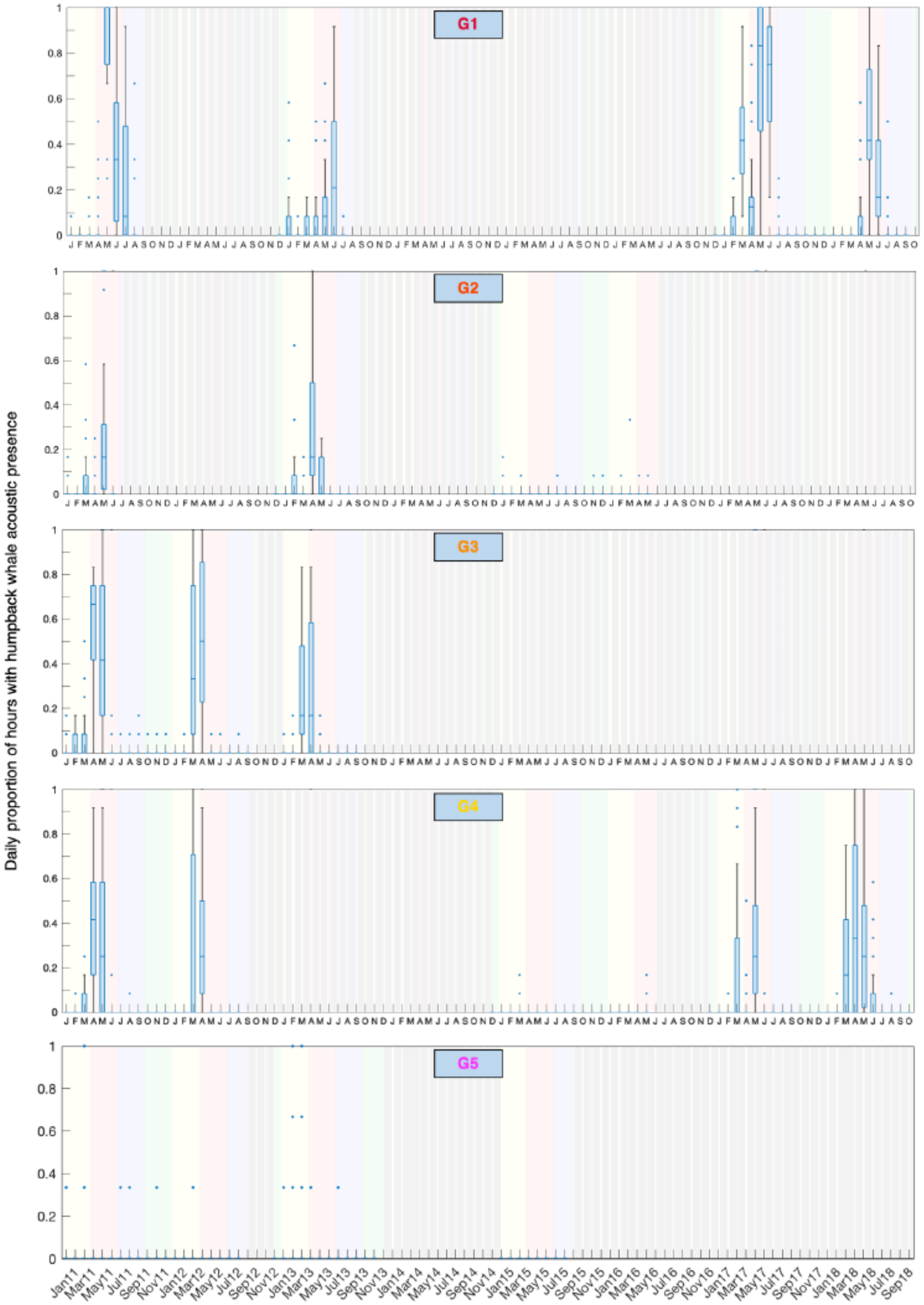

Figure 6. Boxplots of daily proportions of hours with humpback whale acoustic presence from the five recording positions (G1-G5) on the Greenwich Meridian displayed per month from December 2010 until September 2018

(center line, median; box limits, upper and lower quartiles; whiskers, 1.5x interquartile range; points, outliers). Grey bars represent months without recording data, yellow, red, blue and green shades indicate summer, fall, winter and spring seasons, respectively. *S*ingle points indicate single daily observations of humpback whale acoustic presence.

Supplementary Note 3

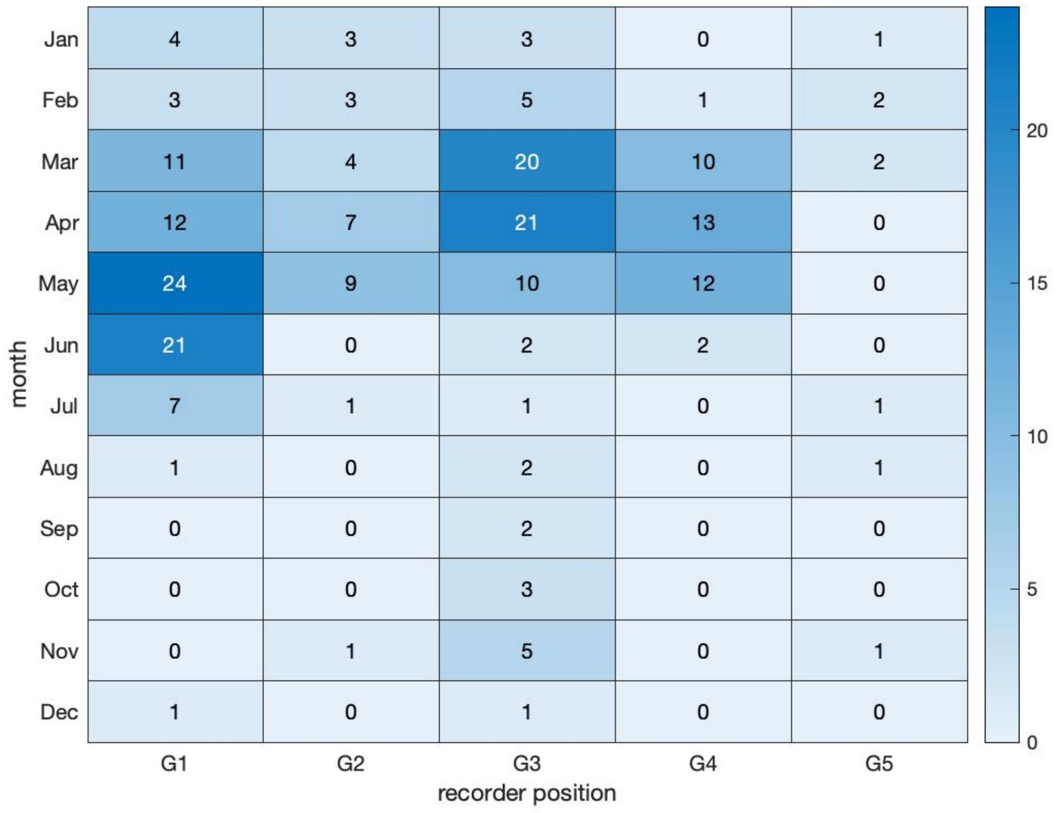

Figure 7. Heatmap showing the average number of days with humpback whale acoustic presence for all months for recording stations G1-5. Darker colors indicate higher presence, numbers in cells represent the average number of days with presence per month per recording station.

Supplementary References

1 Baumgartner, M. F. & Mussoline, S. E. A generalized baleen whale call detection and classification system. *J Acoust Soc Am* **129**, 2889-2902, doi:10.1121/1.3562166 (2011).

2 Schall, E. & Van Opzeeland, I. Calls produced by Ecotype C killer whales (*Orcinus orca*) off the Eckstrom iceshelf, Antarctica. *Aquatic Mammals* **43**, 117-126, doi:10.1578/AM.43.2.2017.117 (2017).

3 Van Opzeeland, I. *et al.* Acoustic ecology of Antarctic pinnipeds. *Marine Ecology Progress Series* **414**, 267-291, doi:10.3354/meps08683 (2010).

4 Risch, D. *et al.* Mysterious bio-duck sound attributed to the Antarctic minke whale (Balaenoptera bonaerensis). *Biol Lett* **10**, 20140175, doi:10.1098/rsbl.2014.0175 (2014).

5 Klinck, H. *et al.* Long-range underwater vocalizations of the crabeater seal (*Lobodon carcinophaga*). *The Journal of the Acoustical Society of America* **128**, 474-479, doi:10.1121/1.3442362 (2010).

6 Ou, H., Au, W. W. L., Van Parijs, S., Oleson, E. M. & Rankin, S. Discrimination of frequency-modulated Baleen whale downsweep calls with overlapping frequencies. *Journal of the Acoustical Society of America* **137**, 3024-3032, doi:10.1121/1.4919304 (2015).

7 Baumgartner, M. F. *et al.* Low frequency vocalizations attributed to sei whales (*Balaenoptera borealis*). *Journal of the Acoustical Society of America* **124**, 1339-1349 (2008).

8 Edds-Walton, P. L. Acoustic communication signals of mysticete whales. *Bioacustics* **8**, 47-60 (1997).

9 Powers, D. M. Evaluation: from precision, recall and F-measure to ROC, informedness, markedness and correlation. *Journal of Machine Learning Technologies* (2011).

10 Dunlop, R. A., Cato, D. H. & Noad, M. J. Non-song acoustic communication in migrating humpback whales (*Megaptera novaeangliae*). *Marine Mammal Science* **24**, 613-629, doi:10.1111/j.1748-7692.2008.00208.x (2008).

11 Magnúsdóttir, E. E. & Lim, R. Subarctic singers: Humpback whale (Megaptera novaeangliae) song structure and progression from an Icelandic feeding ground during winter. *PloS one* **14**, e0210057 (2019).
