# Supplementary material for: Multi-year presence of humpback whales in the Atlantic sector of the Southern Ocean but not during El Niño
Source: Commun Biol. 2021 Jun 25;4:790. doi: 10.1038/s42003-021-02332-6 (PMC8233365; doi:10.1038/s42003-021-02332-6)
Supplement: Supplementary file 3 — Reporting Summary [file 42003_2021_2332_MOESM3_ESM.pdf]

nature research

Corresponding author(s): Elena Schall

Last updated by author(s): May 4, 2021

Reporting Summary

Nature Research wishes to improve the reproducibility of the work that we publish. This form provides structure for consistency and transparency in reporting. For further information on Nature Research policies, see our Editorial Policies and the Editorial Policy Checklist.

Statistics

For all statistical analyses, confirm that the following items are present in the figure legend, table legend, main text, or Methods section.

n/a	Confirmed	
☐	☒	The exact sample size (n) for each experimental group/condition, given as a discrete number and unit of measurement
☐	☒	A statement on whether measurements were taken from distinct samples or whether the same sample was measured repeatedly
☐	☒	The statistical test(s) used AND whether they are one- or two-sided<br>*Only common tests should be described solely by name; describe more complex techniques in the Methods section.*
☒	☐	A description of all covariates tested
☒	☐	A description of any assumptions or corrections, such as tests of normality and adjustment for multiple comparisons
☒	☐	A full description of the statistical parameters including central tendency (e.g. means) or other basic estimates (e.g. regression coefficient) AND variation (e.g. standard deviation) or associated estimates of uncertainty (e.g. confidence intervals)
☐	☒	For null hypothesis testing, the test statistic (e.g. F, t, r) with confidence intervals, effect sizes, degrees of freedom and P value noted<br>*Give P values as exact values whenever suitable.*
☒	☐	For Bayesian analysis, information on the choice of priors and Markov chain Monte Carlo settings
☒	☐	For hierarchical and complex designs, identification of the appropriate level for tests and full reporting of outcomes
☒	☐	Estimates of effect sizes (e.g. Cohen's d, Pearson's r), indicating how they were calculated

Our web collection on statistics for biologists contains articles on many of the points above.

Software and code

Policy information about availability of computer code

Data collection	no software was used
Data analysis	Acoustic data were analyzed with the Low Frequency Detection and Classification System (http://dcs.whoi.edu/lfdcs_manual/lfdcs_manual.html). The detection output was filtered in MATLAB. The manual post-processing of the detections was conducted in Raven Pro 1.6 (https://ravensoundsoftware.com/software/raven-pro/). Statistical analysis was conducted in R (https://www.R-project.org/).

For manuscripts utilizing custom algorithms or software that are central to the research but not yet described in published literature, software must be made available to editors and reviewers. We strongly encourage code deposition in a community repository (e.g. GitHub). See the Nature Research guidelines for submitting code & software for further information.

Data

Policy information about availability of data

All manuscripts must include a data availability statement. This statement should provide the following information, where applicable:

- Accession codes, unique identifiers, or web links for publicly available datasets
- A list of figures that have associated raw data
- A description of any restrictions on data availability

Acoustic presence data: https://datadryad.org/stash/share/p7jYUrL-GX0It3cDw29k5Ff3HBbhrKNrBxuCO2X7XO0.
Sea ice data: https://www.mathworks.com/matlabcentral/fileexchange/50126-daily-antarctic-sea-ice-concentration
ONI data: https://catalog.data.gov/dataset/climate-prediction-center-cpcoceanic-nino-index
SAM data: https://climatedataguide.ucar.edu/climate-data/marshall-southern-annular-mode-sam-index-station-based

April 2020

Field-specific reporting

Please select the one below that is the best fit for your research. If you are not sure, read the appropriate sections before making your selection.

☐ Life sciences ☐ Behavioural & social sciences ☒ Ecological, evolutionary & environmental sciences

For a reference copy of the document with all sections, see nature.com/documents/nr-reporting-summary-flat.pdf

Ecological, evolutionary & environmental sciences study design

All studies must disclose on these points even when the disclosure is negative.

Study description	Passive acoustic data were collected in the Southern Ocean with autonomous mooring systems. Recorders record various sound sources, but in this study only the vocalizations of humpback whales were considered.
Research sample	5 recording positions between 2010 and 2018
Sampling strategy	Data collection depends on difficult logistics and problematic recovery of moorings in the Southern Ocean. All available data were included in the study.
Data collection	Stefanie Spiesecke prepared the recording devices for installation and mooring teams of the RV Polarstern deployed and recovered the moorings.
Timing and spatial scale	G1: 2010-12-11 to 2011-05-21; 2011-05-30 to 2011-05-30; 2011-06-14 to 2011-08-22; 2012-12-11 to 2013-07-13; 2016-12-22 to 2018-09-18;<br>G2: 2010-12-15 to 2011-06-18; 2012-12-14 to 2013-08-02; 2014-12-16 to 2016-05-19;<br>G3: 2010-12-16 to 2012-04-13; 2012-05-06 to 2012-09-17; 2013-01-07 to 2013-09-27;<br>G4: 2010-12-17 to 2012-02-05; 2012-02-28 to 2012-07-30; 2012-08-04 to 2012-08-09; 2012-08-11 to 2012-08-14; 2014-12-18 to 2016-05-28; 2016-12-26 to 2018-10-28;<br>G5: 2010-12-18 to 2012-05-09; 2012-06-01 to 2012-08-10; 2012-12-17 to 2013-05-28; 2013-06-19 to 2013-11-13; 2015-01-08 to 2015-01-26; 2015-02-14 to 2015-02-21; 2015-03-04 to 2015-08-24;<br>The acoustic range of the recording devices was estimated at 50km.
Data exclusions	no data were excluded
Reproducibility	no experiments were conducted, acoustic and statistical analysis is reproducible
Randomization	data were not grouped
Blinding	data analysis was not blinded but data were analyzed by multiple experienced and unexperienced analysts independently.

Did the study involve field work? ☒ Yes ☐ No

Field work, collection and transport

Field conditions	Southern Ocean climatic conditions: Varying among storms, heavy sea ice, calm sea.
Location	G1: 59 2.82 °S; 000 5.78 °E; 1007-1070m<br>G2: 63 59.85 °S; 000 1.84 °E; 970-1007m<br>G3: 66 2.01 °S; 000 3.12 °E ; 934-949m<br>G4: 66 30.71 °S; 000 1.51 °E; 859-1083m<br>G5: 68 59.94 °S; 000 4.38 °E; 958-999m
Access & import/export	UBA (Umwelt Bundesamt) permits for RV Polarstern operation and mooring installation:<br>I 3.5 – 94003-3/271<br>I 3.5 – 94003-3/286<br>I 2.4 – 94003-3/271<br>I 2.4 – 94003-3/255<br>II 2.8 – 94003-3/324<br>II 2.8 – 94003-3/385<br>II 2.8 – 94033/137
Disturbance	RV Polarstern creates disturbance by generation of noise in the Southern Ocean ecosystem, however the ship is always maneuvered in a direct track to minimize disturbance.

Reporting for specific materials, systems and methods

We require information from authors about some types of materials, experimental systems and methods used in many studies. Here, indicate whether each material, system or method listed is relevant to your study. If you are not sure if a list item applies to your research, read the appropriate section before selecting a response.

Materials & experimental systems

n/a	Involved in the study
☒	☐ Antibodies
☒	☐ Eukaryotic cell lines
☒	☐ Palaeontology and archaeology
☒	☐ Animals and other organisms
☒	☐ Human research participants
☒	☐ Clinical data
☒	☐ Dual use research of concern

Methods

n/a	Involved in the study
☒	☐ ChIP-seq
☒	☐ Flow cytometry
☒	☐ MRI-based neuroimaging
